# Long-term ocean acidification trends in coastal waters around Japan

**DOI:** 10.1038/s41598-021-84657-0

**Published:** 2021-03-03

**Authors:** Hiroshi Ishida, Ryosuke S. Isono, Jun Kita, Yutaka W. Watanabe

**Affiliations:** 1 Central Laboratory, Marine Ecology Research Institute, Chiba, 299-5105 Japan; 2 Marine Ecology Research Institute, Tokyo, 162-0801 Japan; 3 Faculty of Environmental Earth Science, Hokkaido University, Sapporo, 060-0810 Japan

**Keywords:** Climate sciences, Environmental sciences, Ocean sciences

## Abstract

This study examines long-term ocean pH data to evaluate ocean acidification (OA) trends at two coastal research institutions located on the Sea of Japan and the Pacific Ocean. These laboratories are located away from the influences of large rivers and major industrial activity. Measurements were performed daily for the past 30 years (1980s–2010s). The average annual ocean pH for both sites showed generally negative trends. These trends were – 0.0032 and – 0.0068 year^–1^ (*p* < 0.001) at the Sea of Japan and Pacific Ocean sites, respectively. The trends were superimposed onto approximately 10-year oscillations, which appear to synchronize with the ocean current periodicity. At the Sea of Japan site, the ocean pH in the summer was higher, and the rate of OA was higher than during other seasons. Our results suggest that seasonality and ocean currents influence OA in the coastal areas of open oceans and can affect the coastal regions of marginal seas.

## Introduction

Ocean acidification (OA) is a global environmental problem^1^_,_ but spatiotemporal variations in coastal water acidification are poorly understood. Previous studies have recognized OA as a problem caused by the ocean absorption of anthropogenic CO_2_ released into the atmosphere^2,3^. The progression of OA has been analyzed from several long-term time-series datasets of open ocean observations^4–9^. Time series data on global ocean carbon chemistry that span the past several decades show that ocean surface acidification exhibits a linearly increasing trend, with an average pH decrease rate of 0.0013 ± 0.0003 to 0.0026 ± 0.0006 year^–1^^7^. Open ocean data continue to be collected, but studies usually do not focus on coastal areas. Therefore, understanding the OA trends in coastal waters due to numerous fisheries and other human activities prevalent along the coast is important based on the collection and analysis of pH data, along with water temperature and salinity measurements. Similarly, appropriate investigation of OA in coastal areas requires long-term data that account for decadal periodicities, i.e., approximately 20 years^10^ as reported for the North Pacific.

In this study, we examined long-term pH data from two coastal institutes located on the Sea of Japan and the Pacific Ocean collected over 30 years using identical measuring techniques. We then assessed whether OA has progressed in the two coastal waters.

## Methods

### Sampling

**Figure 1:**
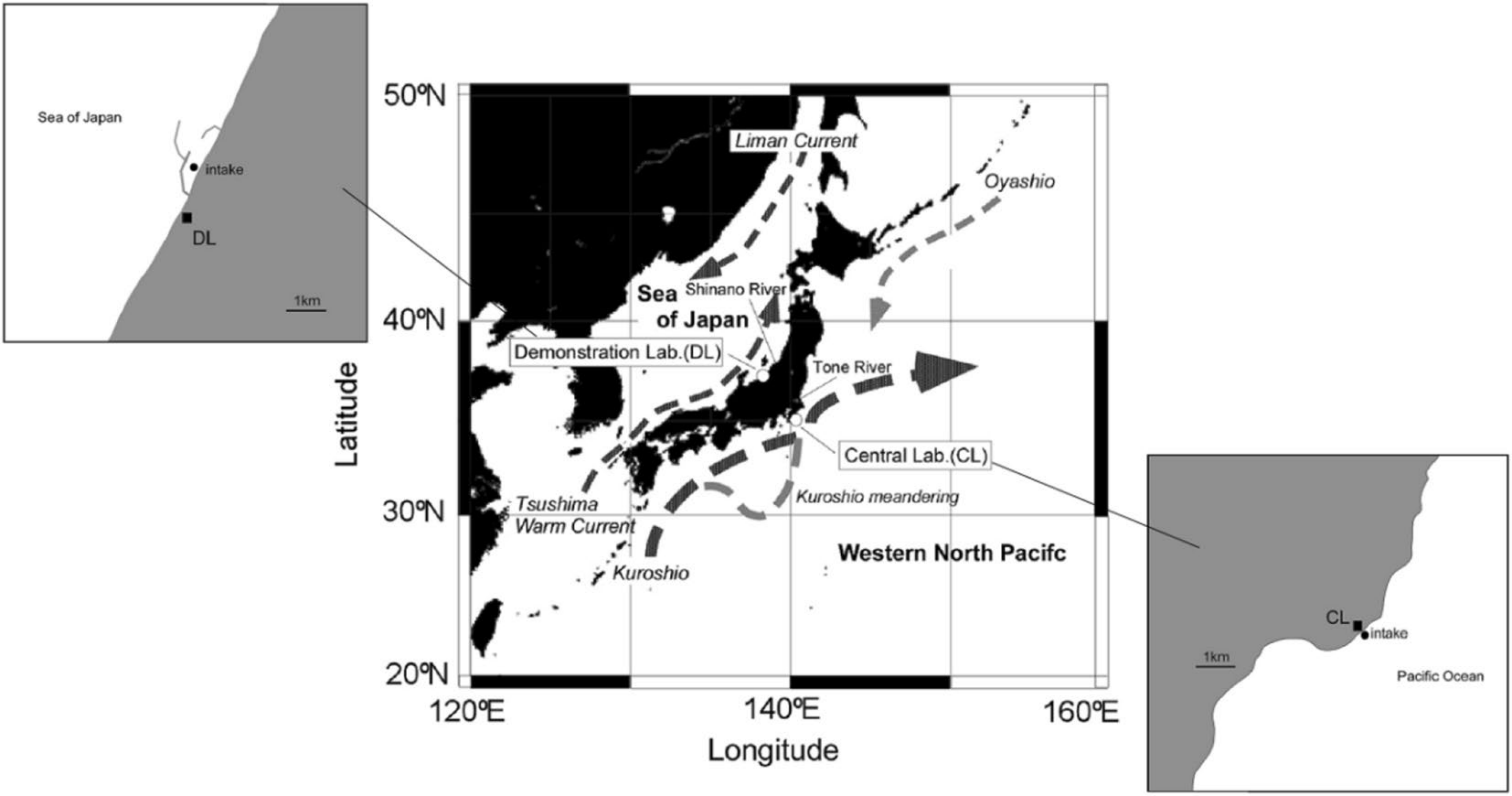
Location of the oceanic pH monitoring sites/laboratories on the Sea of Japan and North Pacific coasts. This figure also shows the major rivers (Shinano and Tone) that are closest to each laboratory and the main oceanic currents that surround Japan.

We monitored the ocean pH daily at two coastal laboratories, i.e., the Demonstration Laboratory (DL) and Central Laboratory (CL), which were established on the Sea of Japan and the Pacific Ocean, respectively, by the Marine Ecology Research Institute of Japan (Fig. 1). These laboratories are located more than 70 km away from major large rivers.

The DL is located in Niigata Prefecture on the coast along the Sea of Japan. Surface sea water samples were collected inside the harbor at the Kashiwazaki-Kariwa Nuclear Power Plant of the Tokyo Electric Power Company Holdings (37°25.5′N, 138°35.4′E) at a rate of 200 m^3^ h^–1^ using a sea water pump. The harbor is a shallow bay on the landward side, created via the installation of a breakwater with a wide opening to the open sea. The distance from the intake to the sampling port was approximately 1 km, with a residence time of approximately 22 min.

The CL is located in Chiba Prefecture on the Pacific coast (35°10.9′N, 140°22.8′E). Here, sea water samples were collected at an approximate water depth of 10 m at a rate of 75 m^3^ h^–1^. The distance from the intake to the sampling port was 350 m, with a residence time of approximately 18 min. Neither sampling system included a filtration tank.

### Measurement

The pH data were obtained at approximately 10:00 daily from January 1992 for the DL and from October 1982 for the CL. The water temperature was also measured simultaneously with the pH measurements.

The pH sensors used were glass electrodes/reference electrodes manufactured by Mettler-Toledo, Inc., which are still in use at both laboratories. The pH was measured until stable by immersing the pH glass electrode while gently stirring the sea water sample in a beaker based on the ISO 10523:2008 standard. Before observation, the pH sensor was calibrated using the following two and/or three different standard buffer solutions: Phthalate pH Standard Solution (pH 4.01 at 25 °C), Phosphate pH Standard Equimolal Solution (pH 6.86 at 25 °C), and Tetraborate pH Standard Solution (pH 9.18 at 25 °C). The standard solution used freshwater as a solvent.

Sea water samples collected at the same time as the pH sample were analyzed for salinity using an Autosal calibrated with the International Association for the Physical Sciences of the Oceans standard sea water.

### Time series analysis of pH data

#### Trend

The annual periodic component was fitted with a sine curve, and its value was removed from the raw data. We then averaged each time series dataset for each year and calculated the standard errors. Trends were defined and examined as follows.

– The average value of the data for each year was used as the representative value for each year.
– The representative value is an independent variable that does not affect the other representative values because the data for one year are averaged, and the data are no longer continuous in time.
– With the survey year as the explanatory variable and each dataset as the objective variable, the data were arranged in chronological order, and linear regression was performed via the ordinary least squares method.
– The slope was used as the annual variation value; there was a significant annual variation when the correlation coefficient, R, was 0.4 or more and the *p*-value was less than 0.001.

#### Oscillations

The pH has a clear bidecadal oscillation superimposed on the linear trends for the subarctic North Pacific open ocean over the past 50 years^11^. Therefore, we attempted to evaluate whether these changes are also characterized by decadal oscillations in coastal areas. To understand decadal pH oscillations, the Fourier sine expansion was applied to the pH time-series datasets from the past 30 years.

The parameters for the Fourier sine expansion included: X = – a · y + b + c · sin {2π(y – d)/e}, where “X” refers to one parameter within the pH, “y” is the calendar year, and “a,” “b,” “c,” “d,” and “e” are constants. In other words, X is the long-term linear trend component plus the decadal oscillation component^12^.

#### Seasonality

To examine and clarify recent seasonal changes in the pH, we divided these pH time-series into four seasonal periods (March–May, spring; June–August, summer; September–November, autumn; and December–February, winter), and then determined a seasonally averaged value for each parameter. The analysis was performed in the same way as the trend analysis described above.

### pH data treatment

We considered water temperature corrections and scale conversion to compare the mean annual oceanic pH time-series for the observed and expected values for the coastal region of the Sea of Japan and North Pacific. To remove the influence of water temperature, the pH data were normalized at 25 °C^13^. The pH, measured by the standard solution prepared with pure water, was termed the pH measured on the NBS scale. As OA has been considered at the total scale, we converted the NBS scale to the total scale for convenience^14^. Data treated with this technique were designated as pH_25T_.

## Results and discussion

### Long-term trend and oscillations

**Figure 2:**
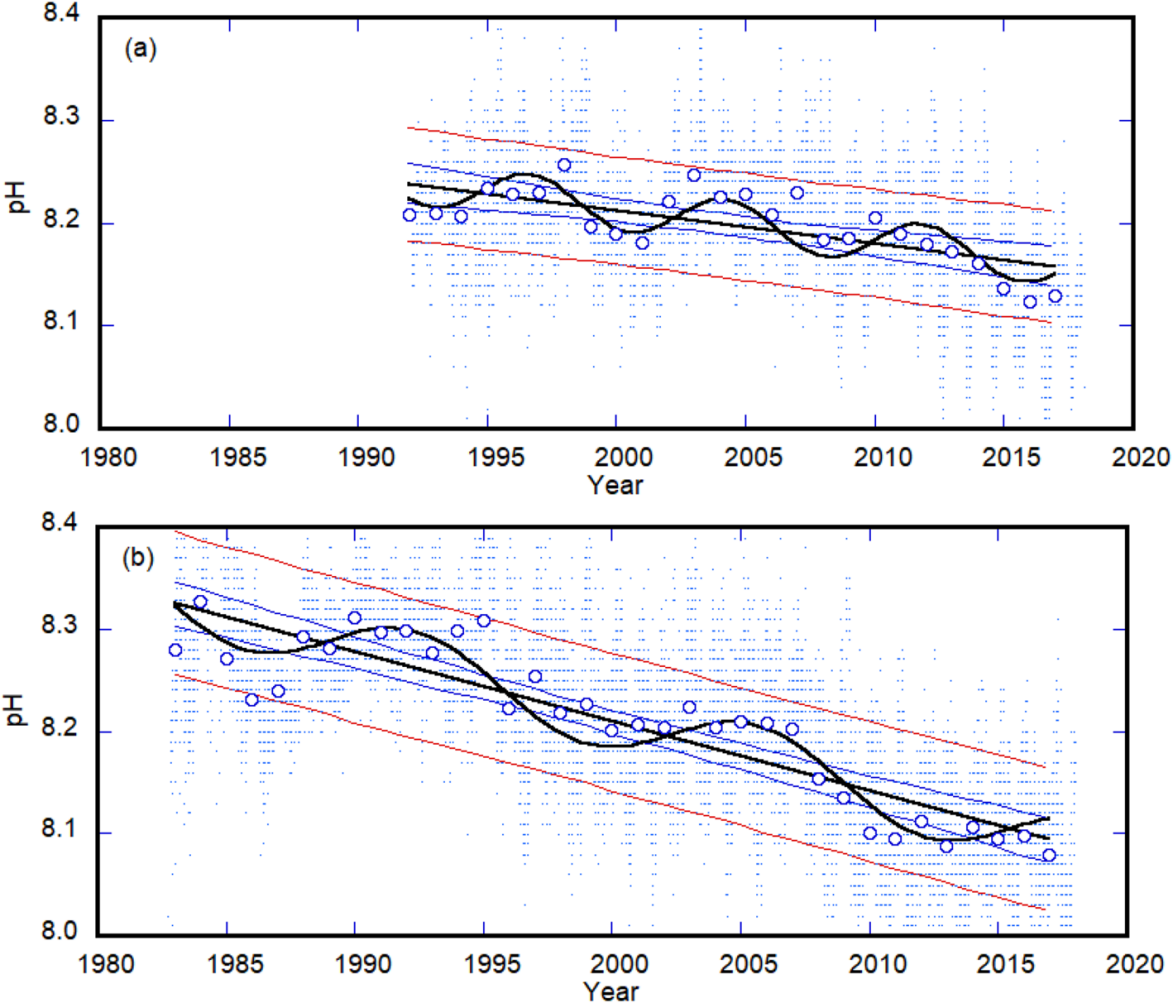
Long-term trends in the oceanic pH in the coastal regions of the Sea of Japan and North Pacific. The Demonstration Laboratory (DL) on the coast of the Sea of Japan **(a)** and Central Laboratory (CL) on the coast of the Pacific Ocean **(b)**. Background dots indicate data. Blue circles show the annual averages. Black straight lines show linear trends while sine curves show decadal oscillations. Blue and red straight lines show the 95% confidence level and prediction intervals of the predicted values, respectively. Table 1 summarizes the trend slopes, acidification rates, statistical *p*-values, and correlation coefficients.

The average annual pH for measurements at the DL and CL had linearly decreasing trends of 0.0032 year^–1^ (*p* < 0.001) and 0.0068 year^–1^ (*p* < 0.001), respectively. The acidification rate at the CL was generally significantly higher than that at the DL (Fig. 2 and Table 1). The acidification rates observed at both laboratories were higher than that of the open ocean (0.0013 to 0.0026 year^–1^ as a linearly decreasing trend)^7^.

**Table 1:** Linear trends and oscillations in the ocean pH for the coastal regions of the Sea of Japan and North Pacific.

Location	Slope (ΔpH year^–1^)					Oscillatory (year)
	Annual average	Seasonal				
		Spring (March to May)	Summer (June to August)	Autumn (September to November)	Winter (December to February)	
Demonstration Laboratory (Sea of Japan)	– 0.0032 (0.71)	– 0.0022 (0.51)	– 0.0060 (0.80)	– 0.0036 (0.72)	*– 0.0013 (0.37)*	7 (0.84)
Central Laboratory (Pacific Ocean)	– 0.0068 (0.89)	– 0.0062 (0.87)	– 0.0068 (0.89)	– 0.0071 (0.88)	– 0.0066 (0.83)	13 (0.95)

The value in the parentheses shows the correlation coefficient: R.

*p*-value of the slope was < 0.001, except for italics number.

The pH at the DL and CL had periodicities of approximately 7 (*p* < 0.01) and 13 years (*p* < 0.01), respectively (Fig. 2 and Table 1). Previous studies have reported long-term biochemical fluctuations in the pelagic regions of the North Pacific and Sea of Japan, with periodicities of 18 to 20 years^12,15^. The periodicities observed in this study, however, were shorter than those previously reported. In the Sea of Japan, the Tsushima warm current (Fig. 1) has a periodicity of 5 to 9 years and a high salinity water temperature anomaly^16^, whereas at the Pacific site, the Kuroshio meander (Fig. 1) has occurred every 12 to 15 years since the 1990s^17^. Although we do not understand the oceanographic mechanism that produces these periodicities, these cyclic changes in the ocean current environment may influence OA in surrounding coastal areas.

The intake sea water in both laboratories is affected by currents and tides because the intake location is not a closed bay (Fig. 1). The pH is thought to be affected by tidal changes and water masses, such that their short-term effects on the long-term trend analysis are of concern. However, daily fluctuations in the water mass (salinity, water temperature, and density) at the facility were not unique, as they may or may not correspond to tides (Supplementary Fig. S1). Furthermore, seasonal fluctuations were greater than daily fluctuations (Supplementary Fig. S1, S2). For the salinity, the effects of rainfall, snow cover, and snowmelt can be considered at both laboratories. In addition, the CL may have been affected by freshwater supplied from the Tone River. However, these factors, which cause a decrease in the salinity, are considered to be seasonal events (Supplementary Fig. S3). Our analysis averaged the data obtained once daily for one year. Although we cannot state that there is no relationship between the tide, water mass, and pH, our long-term analysis suggests that these relationships do not have a significant effect.

### Difference from theoretical ocean acidification

We evaluated by how much the measured OA rates differ from the theoretical OA trend. The theoretical OA rate was estimated from atmospheric *p*CO_2_ time-series data and estimated alkalinity. Using the Mauna Loa CO_2_ data^18^ as a representative atmospheric *p*CO_2_ time-series for the northern hemisphere, we calculated the theoretical pH with the CO2SYS program^19^ at each experimental site. Since 2015, alkalinity measurements were also made at the DL. Salinity measurements are performed at the DL and CL simultaneously with the pH measurements daily. The estimated alkalinity values for the DL were calculated using the following equation, with the salinity values measured for the same sampling point:

${\text{Estimated alkalinity }}(\upmu {\text{mol kg}}^{{-1}} ) \, = { 44}.{\text{89 Salinity }}\left( {{\text{PSU}}} \right) \, + { 699}.{72 }\left( {{\text{R }} = \, 0.{8}} \right).$The estimated alkalinity of CL samples was calculated with the salinity and temperature data obtained at the time of the pH measurements^20^.

**Figure 3:**
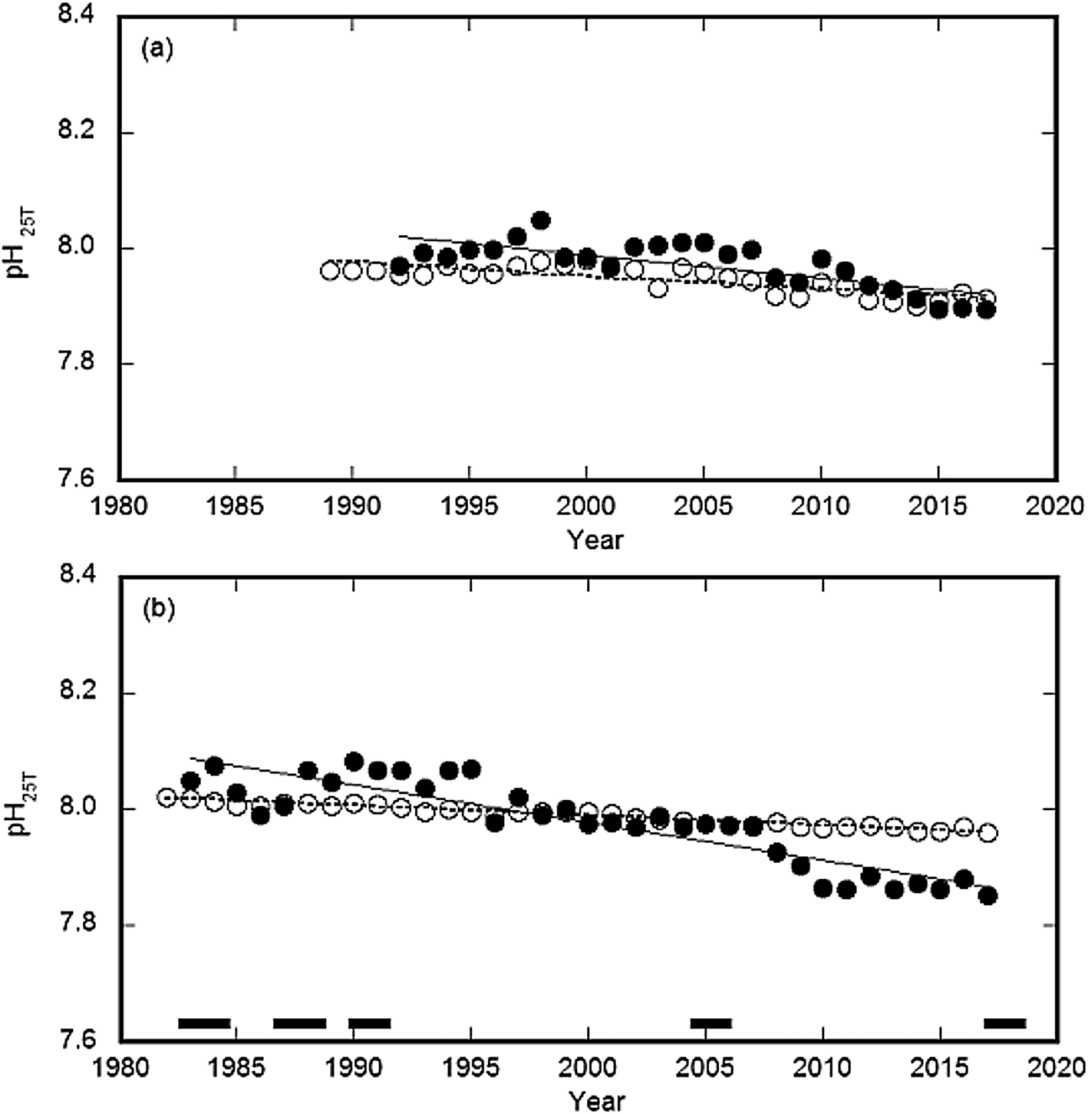
Comparison between the mean annual oceanic pH_25T_ time-series for the observed and expected values for the coastal regions of the Sea of Japan and North Pacific. The Demonstration Laboratory (DL) on the Sea of Japan coast **(a)** and Central Laboratory (CL) on the Pacific Ocean coast **(b)**. White circles show theoretical values and black circles show annual observation averages. The dashed line indicates the linear trends in the theoretical variability for the DL and CL; these decreasing trends are 0.0022 and 0.0016 year^–1^, respectively. The solid line shows the linear trend in the annual observation averages for the DL and CL; these slopes are identical to the annual averages, which are – 0.0037 and – 0.0064 year^–1^, respectively. The thick solid lines show the periods during which Kuroshio meandering was observed.

The theoretical acidification rates, as decreasing pH_25T_ trends, were estimated as 0.0022 and 0.0016 year^–1^ for the DL and CL, respectively (Fig. 3). The average annual slope at each site was 2 to 4 times higher than that for the theoretical values (Fig. 3), which suggests that, for the coastal waters at the DL and CL, there are factors that promote OA other than atmospheric CO_2_.

An increase in the water temperature may be a direct factor in lowering the pH. Several studies in the Japan Sea have reported significant increasing water temperature trends at depths lower than the mixed layer^15,21^. However, we did not find this increasing trend in the DL, potentially because of surface water (Supplementary Fig. S4). No such increasing trend was found in the CL (Supplementary Fig. S4), consistent with an insignificant increasing sea surface water temperature in the North Pacific^22^. In contrast, in our survey over the last 30 years, there was a trend only in the salinity at the DL in the water masses (Supplementary Fig. S4). A previous study reported a periodicity of 5 to 9 years in the high-salinity water distribution area of the Sea of Japan^16^, which is consistent with the periodicity of the pH-decreasing tendency for the DL. Salinity and/or factors that cause changes in the salinity may affect OA. For example, rivers and groundwater can supply chemical substances that lower the pH, along with freshwater.

Eutrophication may promote OA on the coast^23^. Previous studies have suggested that factors other than atmospheric CO_2_ alone may promote the acidification rate in coastal areas^24,25^. Since 1990 on the Pacific coast, Kuroshio meandering has occurred every 12 to 15 years^17^. The pH_25T_ values for waters from the CL appeared to decline several years after the occurrence of a Kuroshio meander (Fig. 3b). Previous studies have noted that the primary productivity along the coast improves when the Kuroshio meanders^26^. A possible mechanism may exist where organic matter production increases during the meander and accumulates in the coastal waters surrounding the CL, which enriches the organic matter in the surface sediments and leads to the release of CO_2_ via decomposition, followed by a decrease in the pH_25T_. Changes in the biophysical and chemical environment surrounding the CL, possibly due to changes in the Kuroshio Current axis, accompany changes in the pH of coastal waters from the CL.

### Seasonal fluctuations

**Figure 4:**
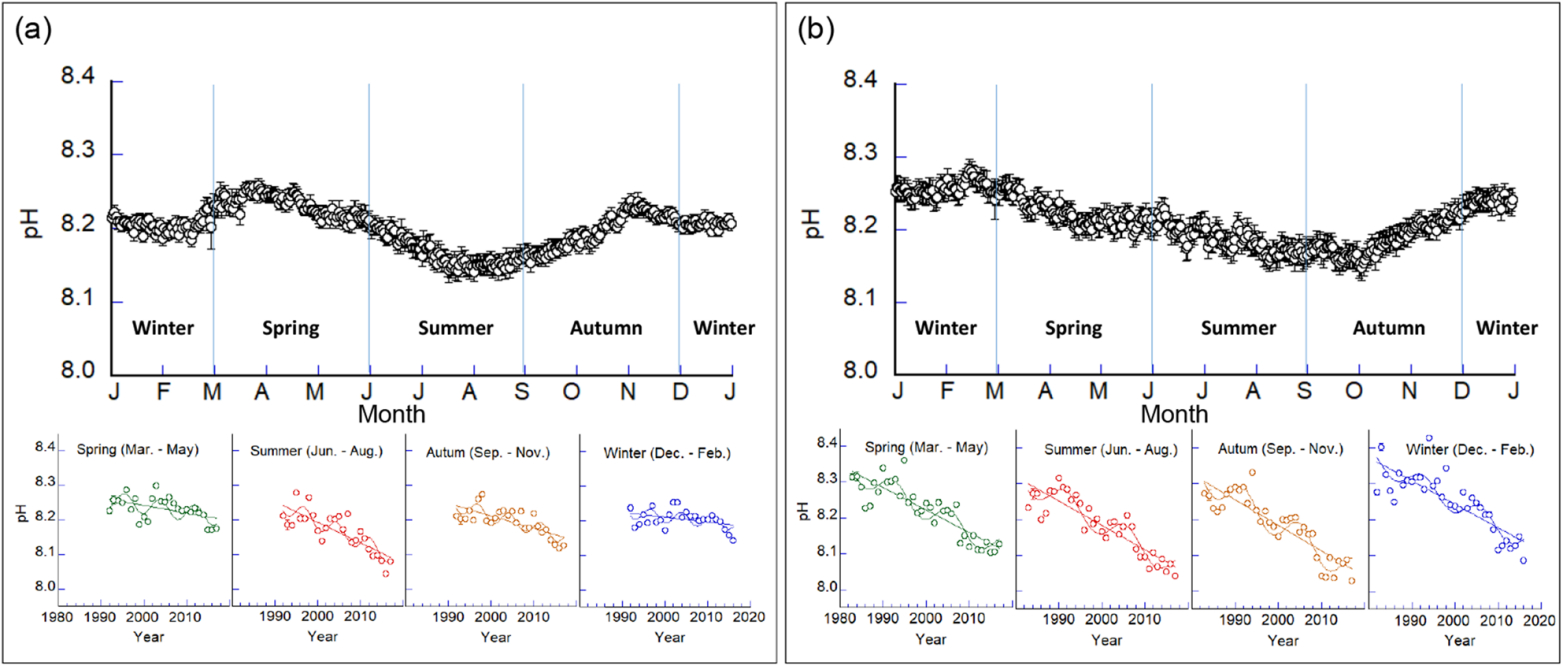
Seasonal variations and long-term trends in the pH by season for the coastal region of the Sea of Japan and North Pacific. The Demonstration Laboratory (DL) on the coast of the Sea of Japan **(a)** and Central Laboratory (CL) on the coast of the Pacific Ocean **(b)**. The upper figure shows daily averaged values, where the error bars represent the standard errors. The lower figure shows the long-term trends in the oceanic pH by season. Straight lines show linear trends while sine curves show decadal oscillations. Table 1 summarizes the trend slopes, acidification rates, statistical *p*-values, and correlation coefficients.

Both laboratories are characterized by seasonal changes in the ocean pH. For the DL, the mean daily pH varies from 8.14 to 8.26 annually, with a lower pH in the summer (June to August). In contrast, the mean daily pH at the CL fluctuates from 8.15 to 8.28 throughout the year, and the pH is lowest in autumn (September to November) (Fig. 4). To clarify the long-term changes in the pH for each season, we divided the annual data into four seasons and evaluated the long-term seasonal pH trends. Both laboratories demonstrated a long-term decline in the pH with each season. The DL is characterized by declining trends in the pH of 0.0022, 0.0060, 0.0036, and 0.0013 year^–1^ in the spring, summer, autumn, and winter, respectively (*p* < 0.001 except for winter) (Table 1). This demonstrates that the declining rate in the pH varies seasonally, and the rate of decline during the summer is larger than that for the other seasons. For the CL, the rate of decline varies from 0.0062 to 0.0071 year^–1^, which indicates less of a seasonal difference than that for the DL (Table 1). Ocean pH values at the DL and CL were measured from the sea surface water and at depths of 10 m, respectively. The seasonal differences in the acidification rates at the DL and CL are possibly due to differences in their sampling depths. However, both coasts are nearly vertically mixed, except for cases of significant stratification in the summer. Therefore, the water temperature, salinity, and pH of the sea surface and at water depths of 10 m are nearly identical. In other words, the seasonal difference in the acidification rates is indicative of the trends at the DL and CL, except for when vertical stratification in the summer is significant along both coasts. Considering that the acidification rate of the DL (near the sea surface) in summer, when stratification is well developed, is higher than in other seasons, as compared with the CL, the acidification rate near the sea surface may be higher than that at a depth of 10 m in summer at the CL. A previous study pointed out that the effects of OA on marine organisms are strong in the summer and mitigated in winter^24^. The acceleration of acidification on the Sea of Japan side is higher in the summer than in other seasons, suggesting that the effects on the marine organisms inhabiting it may also differ depending on the season.

Based on the results obtained in this study, we conclude that we are unable to explain OA in coastal areas based solely on the increasing concentrations of CO_2_ in the atmosphere, as well as the fact that OA has both regional environmental characteristics and seasonal fluctuations. Whether the DL and CL results indicate typical trends for other coastal areas in the Sea of Japan and Pacific remains unclear. However, the locations of both laboratories were not directly affected by large rivers or industrial activities. In coastal zones that belong to marginal waters and/or that face the open ocean with similar environmental characteristics, acidification rates may vary seasonally and be higher than those for the open ocean. To predict the effects that OA has on marine organisms and their biological adaptability in coastal regions, we must consider the results of appropriate monitoring while attempting to understand the regional environmental characteristics, rather than relying on acidification trends observed in the open ocean.

## Supplementary information

Supplementary figures.

## References

[1] Bellerby RGJ (2017). Ocean acidification without borders. Nat. Clim. Change.

[2] Calderia K, Wickett ME (2003). Anthropogenic carbon and ocean pH. Nature.

[3] Orr JC, Fabry VJ, Aumont O, Bopp L (2005). Anthropogenic ocean acidification over the twenty-first century and its impact on calcifying organisms. Nature.

[4] Gonzalez-Davila M, Santana-Casiano JM, González-dávila E (2007). Interannual variability of the upper ocean carbon cycle in the northeast Atlantic Ocean. Geophys. Res. Lett..

[5] Dore, J. E., Lukas, R., Salder, D. W., Church, M. J. & Karl, D. M. Physical and biogeochemical modulation of ocean acidification in the central North Pacific. *Proc. Natl. Acad. Sci.* **106,** 12 235−12 240 (2009). 10.1073/pnas.0906044106 PMC2716384 19666624

[6] Bates NR (2007). Interannual variability of the ocean CO_2_ sink in the subtropical gyre of the North Atlantic Ocean over the last 2 decades. J. Geophys. Res..

[7] Bates NR, Astor YM, Church MJ, Currie KI (2014). A time-series view of changing surface ocean chemistry due to ocean uptake of anthropogenic CO_2_ and ocean acidification. Oceanography.

[8] Midorikawa, T. et al. Decreasing pH trend estimated from 25-year time series of carbonate parameters in the western North Pacific. *Tellus B Chem. Phys. Meteorol*. **62 (5)**, 649–659, 10.1111/j.1600-0889.2010.00474.x.

[9] Olafsson J (2009). Rate of Iceland Sea acidification from time series measurements. Biogeosciences.

[10] Yasuda, I. Impact of the astronomical lunar 18.6-year tidal cycle on El-Nino and Southern Oscillation. *Sci. Rep.* **8**, 15206 (2018). 10.1038/s41598-018-33526-4 PMC6185980 30315185

[11] Watanabe WY, Li BF, Wakita M (2018). Long-term trends of direct and indirect anthropogenic effects on changes in ocean pH. Geophys. Res. Lett..

[12] Watanabe WY, Ishida H, Nakano T, Nagai N (2005). Spatiotemporal decreases of nutrients and chlorophyll-a in the surface mixed layer of the western North Pacific from 1971 to 2000. J. Oceanogr..

[13] Millero JF (1995). Thermodynamics of the carbon dioxide system in the oceans. Geochim. Cosmochim. Acta.

[14] Millero JF (1979). The thermodynamics of the carbonate system in seawater. Geochim. Cosmochim. Acta.

[15] Watanabe WY, Wakita M, Maeda N, Ono T (2003). Synchronous bidecadal periodic changes of oxygen, phosphate and temperature between the Japan Sea deep water and the North Pacific intermediate water. Geophys. Res. Lett..

[16] Naganuma, K. On the water temperature fluctuation at the representative points in the Japan Sea along the Honshu Coast. *Bull. Jpn. Sea Reg. Fish. Res. Lab.* **29**, 269–282 (1978).

[17] JMA. https://www.data.jma.go.jp/kaiyou/data/shindan/b_2/kuroshio_stream/kuroshio_stream.html.

[18] NOAA Earth System Research Laboratory. ftp://aftp.cmdl.noaa.gov/products/trends/co2/co2_annmean_mlo.txt.

[19] Lewis, E., & Wallace, D. W. R. *Program developed for CO*_*2*_* system calculations, ORNL/CDIAC-105. Oak Ridge: Carbon dioxide information analysis center, Oak Ridge National Laboratory, U.S. Department of Energy* (1998).

[20] Lee K, Tong LT, Millero FJ, Chris S (2006). Global relationships of total alkalinity with salinity and temperature in surface waters of the world’s oceans. Geophys. Res. Lett..

[21] Gamo T (2011). Dissolved oxygen in the bottom water of the Sea of Japan as a sensitive alarm for global climate change. Trends Anal. Chem..

[22] Levitus S, Antonov JI, Boyer TP, Baranova OK (2012). World ocean heat content and thermosteric sea level change (0–2000 m), 1955–2010. Geophys. Res. Lett..

[23] Sunda, W. G., & Cai, W.-J. Eutrophication induced CO_2_-acidification of subsurface coastal waters: Interactive effects of temperature, salinity, and atmospheric PCO_2_. *Environ. Sci. Technol*. **46**(19), 10651–10659 (2012). 10.1021/es300626f 22889106

[24] Jacob C, Duarte CM (2019). Drivers of pH variability in coastal ecosystems. Environ. Sci. Technol..

[25] Kwiatkowski L, Orr JC (2018). Diverging seasonal extremes for ocean acidification during the twenty-first century. Nat. Clim. Change.

[26] *ISEE Newslette*r Vol. 6 (Nagoya University, 2018)*.*

