# Supplementary material for: Long-term ocean acidification trends in coastal waters around Japan
Source: Sci Rep. 2021 Mar 3;11:5052. doi: 10.1038/s41598-021-84657-0 (PMC7930190; doi:10.1038/s41598-021-84657-0)
Supplement: Supplementary file 1 — Supplementary figures. [file 41598_2021_84657_MOESM1_ESM.pdf]

Long-term ocean acidification trends in coastal waters around Japan

Hiroshi Ishida[1*], Ryosuke S. Isono[1], Jun Kita[2], and Yutaka W. Watanabe[3]

[1] Central Laboratory, Marine Ecology Research Institute, Chiba, 299-5105, JAPAN

[2] Head Office, Marine Ecology Research Institute, Tokyo, 162-0801, JAPAN

[3] Faculty of Environmental Earth Science, Hokkaido University, Sapporo, 060-0810, JAPAN

[*] E-mail: h-ishida@kaiseiken.or.jp

Supplementary information

Relationship between pH, tides, and water mass

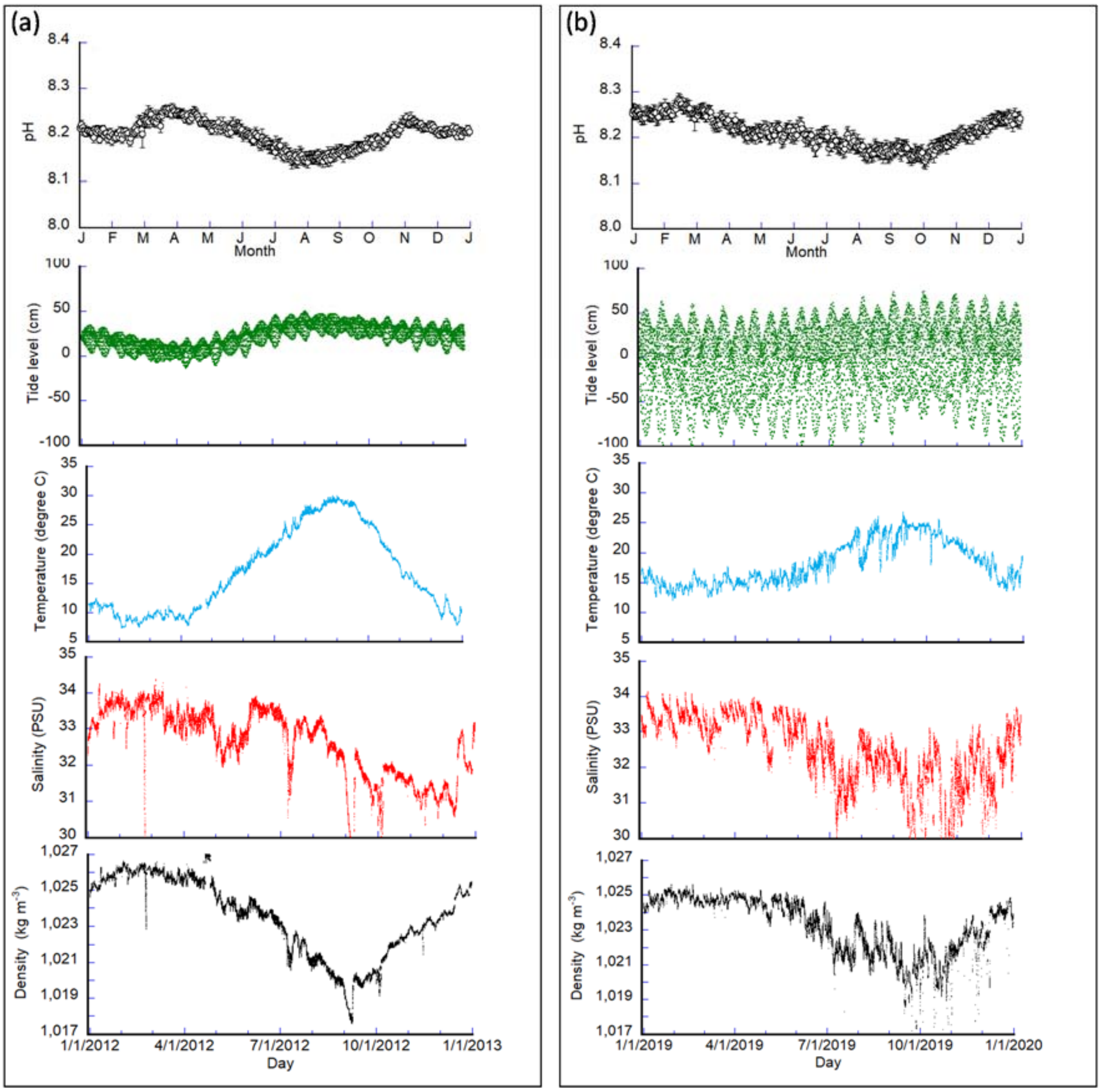

Figure S1. Annual variability in the pH, tides, and water masses for the studied coastal regions of the Sea of Japan and North Pacific. The Demonstration Laboratory (DL) on the coast of the Sea of Japan (a) and Central Laboratory (CL) on the coast of the Pacific Ocean (b). The graphs show the pH, tide level, temperature, salinity, and density. The water mass data were obtained at 10 min intervals, the pH was measured once per day, and tide level data were obtained at 1 h intervals. The tide data for the DL are based on records from Kashiwazaki and the CL tide data are based on records from Katsuura, which is approximately 10 km away. These data were obtained from the Japan Meteorological Agency website (https://www.data.jma.go.jp/gmd/kaiyou/db/tide/sea_lev_var/index_hourly.php). The observation period was 1/1/2012 to 12/21/2012 at DL and 1/1/2019 to 12/312019 at CL.

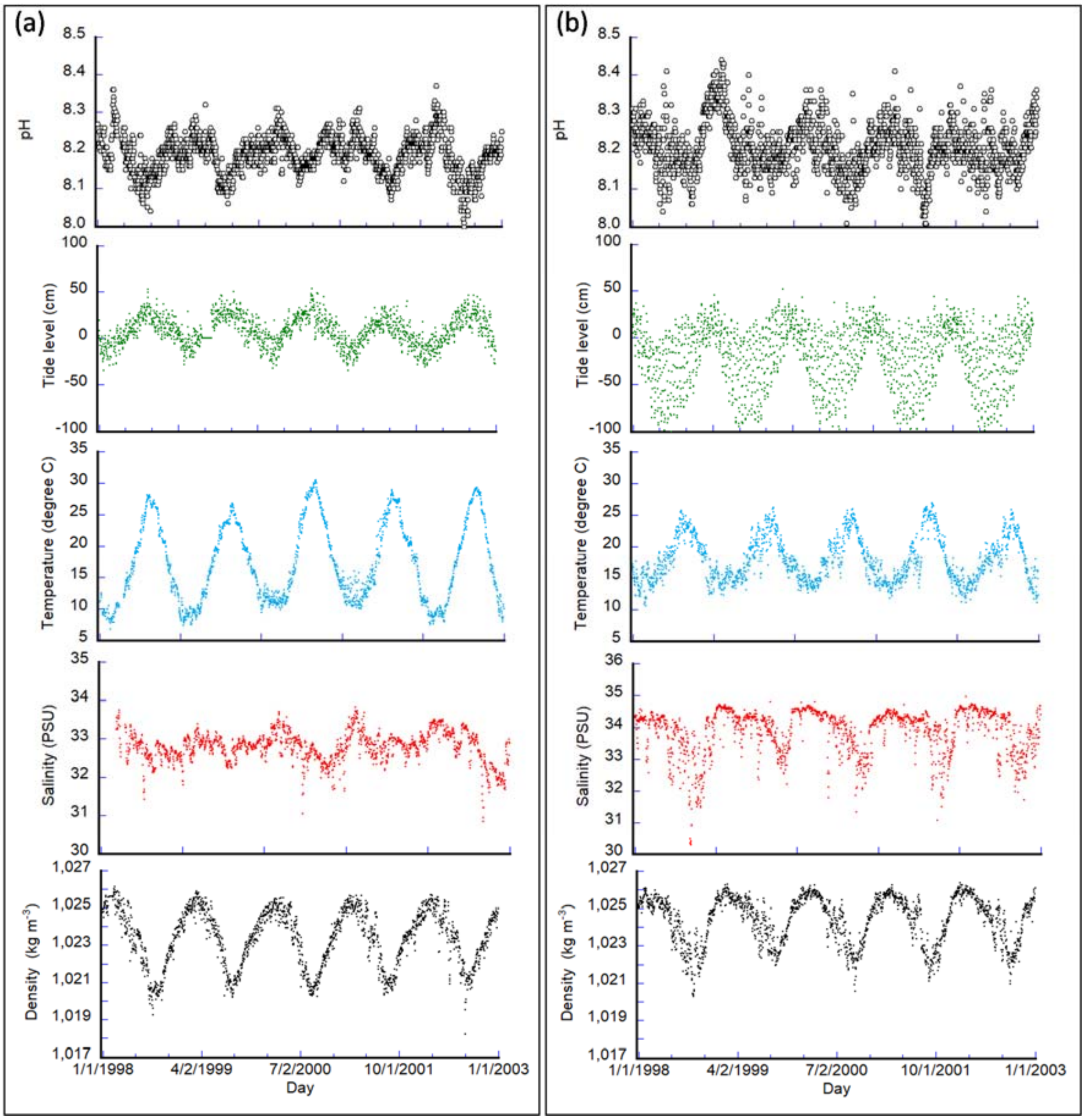

Figure S2. Long-term (5 year) variability pattern of the pH, tides, and water masses for the studied coastal regions of the Sea of Japan and North Pacific. The Demonstration Laboratory (DL) on the coast of the Sea of Japan (a) and Central Laboratory (CL) on the coast of the Pacific Ocean (b). The graphs show the pH, tide level, temperature, salinity, and density. The pH data were obtained at approximately 10:00 daily. The water mass data were measured simultaneously with pH. The long-term tide data for the DL are based on records from Sado, an island approximately 20 km from the DL. For the CL, tide data are based on records from Choshi, which is a city approximately 70 km away from the CL. From each observation, we used data collected at 10:00 each day. Tide level data were obtained from the

Japan Meteorological Agency website.

(https://www.data.jma.go.jp/gmd/kaiyou/db/tide/sea_lev_var/index_hourly.php). The observation period is 1/1/1998 to 12/31/2002. In general, the ebb and flow of water at a given point usually occurs twice a day, and the average cycle from one low tide to the next is approximately 12 h and 25 min. This indicates that the ebb tide is delayed by approximately 50 min every day. The tidal conditions at the time of measurement (approximately 10:00 each day) are not the same; they may be at high tide, at low tide, or heading toward each.

Changes in salinity and weather conditions

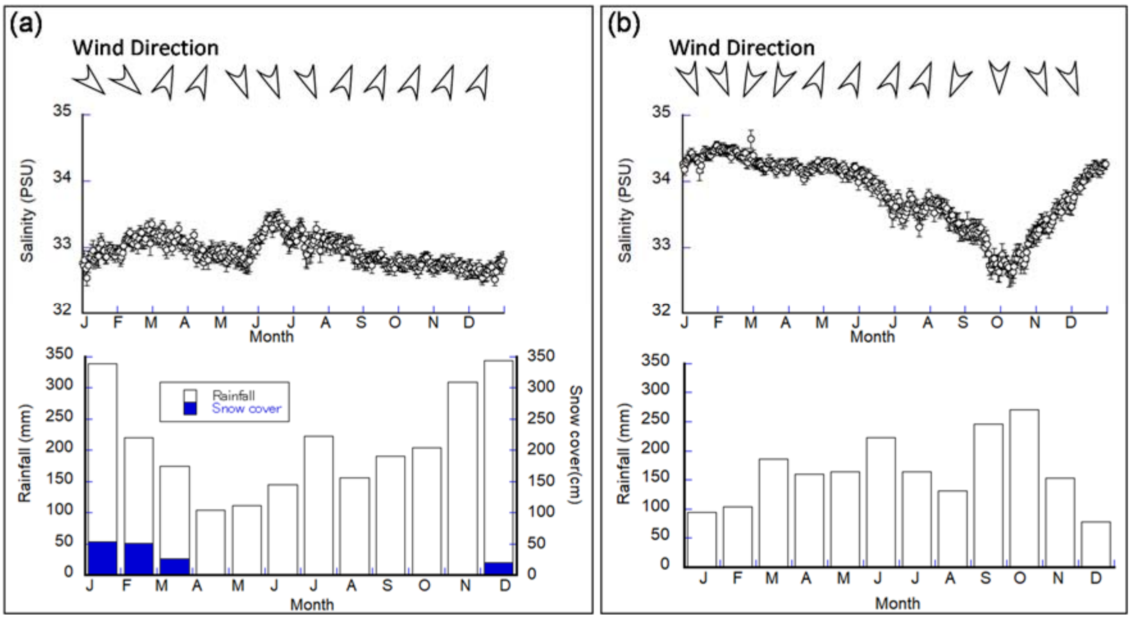

Figure S3. Seasonal fluctuation patterns of the salinity and meteorological conditions for the studied coastal regions of the Sea of Japan and North Pacific. The Demonstration Laboratory (DL) on the coast of the Sea of Japan (a) and Central Laboratory (CL) on the coast of the Pacific Ocean (b). Meteorological data are the monthly average and consider data from Kashiwazaki and Katsuura as the DL and CL. Kashiwazaki is an area with DL. Katsuura is approximately 10 km away from CL. Data were obtained from the Japan Meteorological Agency website (https://www.data.jma.go.jp/obd/stats/etrn/). Salinity decreases in January and June of each year in the DL and around October of each year in the CL. The DL has an influx of freshwater due to rainfall in January and snowmelt in June; the influence of freshwater was considered. The CL experiences significant rainfall in September and October, and there is also an increase in the quantity of freshwater discharged from large rivers on the northwest side. The decreased salinity was assumed to be attributable to the southward movement of freshwater mixed with sea water along the coast due to the north–northeast monsoon.

Long-term trend in water masses

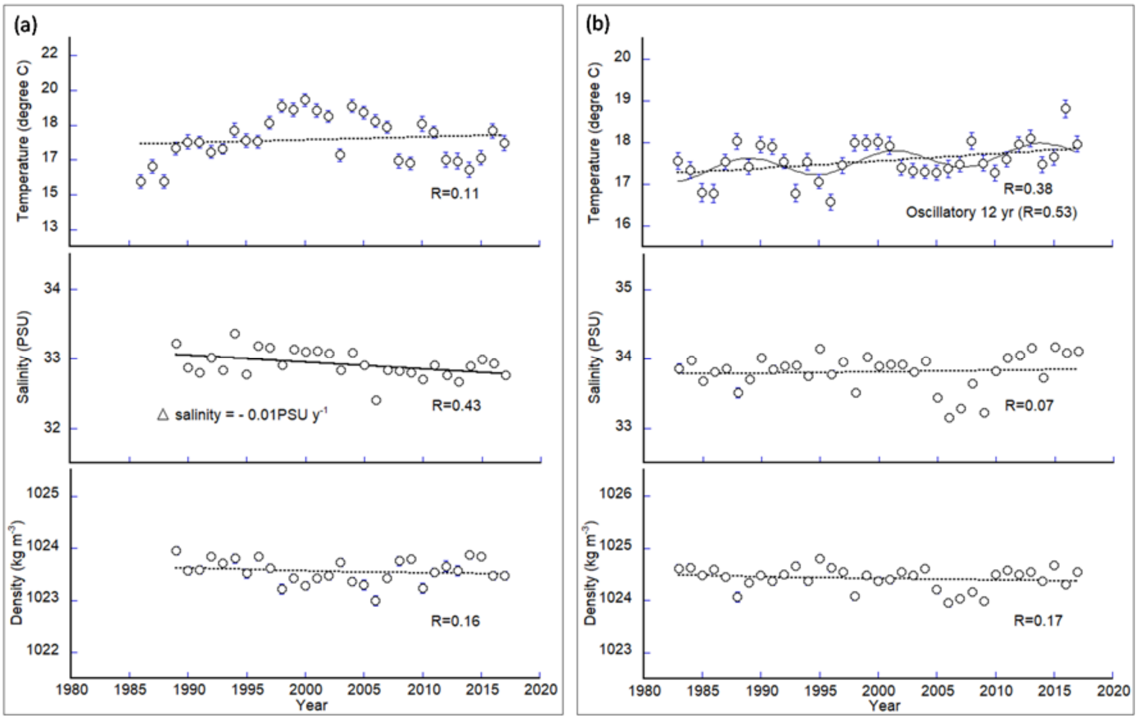

Figure S4. Long-term trends in the annual average of the sea water temperature, salinity, and density for the studied coastal regions of the Sea of Japan and North Pacific. The Demonstration Laboratory (DL) on the coast of the Sea of Japan (a) and Central Laboratory (CL) on the coast of the Pacific Ocean (b). For the salinity at DL, the correlation coefficient (R) was 0.43, as shown by the solid line. The slopes are also shown in the figure, as we assumed that there is a linear tendency. Others are shown by dotted lines, but the correlation coefficient was below 0.4. For the water temperature at CL, oscillations with a period of approximately 12 years were also observed (R = 0.53).
